# Data set of fraction unbound values in the *in vitro* incubations for metabolic studies for better prediction of human clearance

**DOI:** 10.1093/database/baae063

**Published:** 2024-07-24

**Authors:** Laura Krumpholz, Aleksandra Klimczyk, Wiktoria Bieniek, Sebastian Polak, Barbara Wiśniowska

**Affiliations:** Pharmacoepidemiology and Pharmacoeconomics Unit, Faculty of Pharmacy, Jagiellonian University Medical College, Medyczna 9, Street, Kraków 30-688, Poland; Doctoral School in Medical and Health Sciences, Jagiellonian University Medical College, Łazarza Street 16, Kraków 31-530, Poland; Pharmacoepidemiology and Pharmacoeconomics Unit, Faculty of Pharmacy, Jagiellonian University Medical College, Medyczna 9, Street, Kraków 30-688, Poland; Pharmacoepidemiology and Pharmacoeconomics Unit, Faculty of Pharmacy, Jagiellonian University Medical College, Medyczna 9, Street, Kraków 30-688, Poland; Chair of Pharmaceutical Technology and Biopharmaceutics, Faculty of Pharmacy, Jagiellonian University Medical College, Medyczna 9, Street, Krakow 30-688, Poland; Certara UK Ltd (Simcyp Division), 1 Concourse Way, Sheffield S1 2BJ, United Kingdom; Pharmacoepidemiology and Pharmacoeconomics Unit, Faculty of Pharmacy, Jagiellonian University Medical College, Medyczna 9, Street, Kraków 30-688, Poland

## Abstract

*In vitro–in vivo* extrapolation is a commonly applied technique for liver clearance prediction. Various *in vitro* models are available such as hepatocytes, human liver microsomes, or recombinant cytochromes P450. According to the free drug theory, only the unbound fraction (fu) of a chemical can undergo metabolic changes. Therefore, to ensure the reliability of predictions, both specific and nonspecific binding in the model should be accounted. However, the fraction unbound in the experiment is often not reported. The study aimed to provide a detailed repository of the literature data on the compound’s fu value in various *in vitro* systems used for drug metabolism evaluation and corresponding human plasma binding levels. Data on the free fraction in plasma and different *in vitro* models were supplemented with the following information: the experimental method used for the assessment of the degree of drug binding, protein or cell concentration in the incubation, and other experimental conditions, if different from the standard ones, species, reference to the source publication, and the author’s name and date of publication. In total, we collected 129 literature studies on 1425 different compounds. The provided data set can be used as a reference for scientists involved in pharmacokinetic/physiologically based pharmacokinetic modelling as well as researchers interested in Quantitative Structure-Activity Relationship models for the prediction of fraction unbound based on compound structure.

**Database URL**: https://data.mendeley.com/datasets/3bs5526htd/1

## Introduction

The process of developing an innovative drug and introducing it to the market is challenging, time-consuming, expensive, and characterized by poor success rate [1]. Modelling and simulation are the approach taken in an attempt to improve their efficiency and productivity and accelerate drug development [2].

Absorption, distribution, metabolism, and elimination (ADME) properties encompass a range of processes that influence the pharmacokinetics and pharmacodynamics of a drug within the body and, therefore, decide its value. By accurate prediction of these properties, the potential effectiveness, safety, and pharmacokinetic (PK) behaviour of a compound can be assessed, enabling informed decision-making and optimization of drug candidates. This facilitates the selection of candidates with favourable PK profiles for further development stages and aids in identifying potential safety concerns associated with drug candidates before first-in-human studies. Therefore, the prediction of parameters related to the ADME of a compound has a key role in the optimization of that process [3–6].

**Figure 1.**
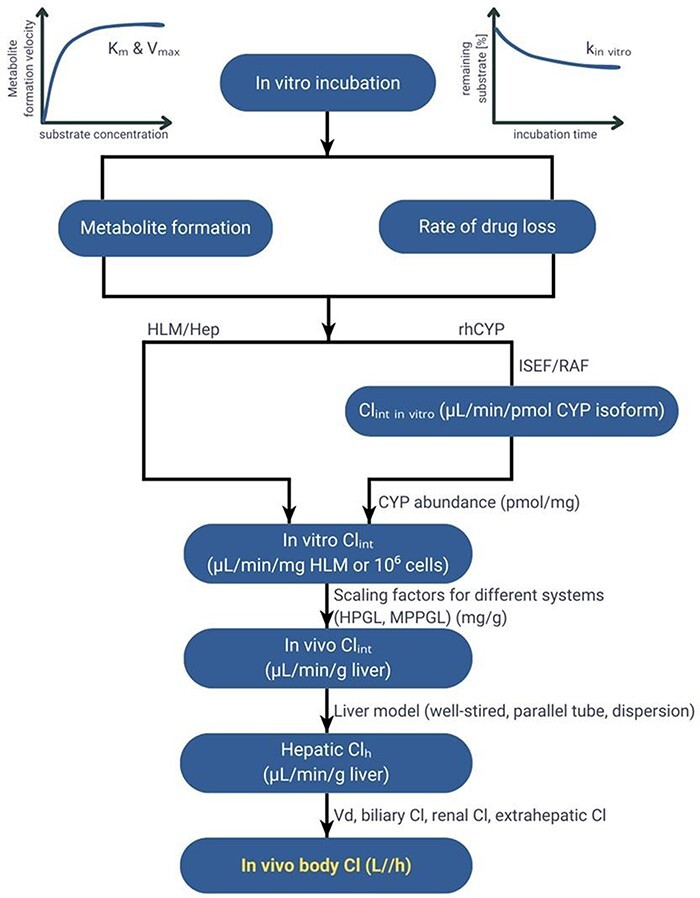
IVIVE process.

Clearance is one of the critically important PK parameters influencing systemic exposure and thus efficacy and safety of the therapy. Therefore, its accurate estimation is one of the most critical tasks in drug development. Various methods have been developed to predict human clearance [7–10]. The most employed approach, an *in vitro*–*in vivo* extrapolation (IVIVE), involves the utilization of experimental *in vitro* metabolic data (Fig. 1). This approach includes the determination of intrinsic clearance of a compound using various *in vitro* systems such as hepatocytes, microsomes, or recombinant enzymes, and its integration with physiological parameters within the mathematical framework to estimate clearance in humans [9]. The intrinsic tissue/organ clearance estimated based on *in vitro* intrinsic clearance value is then incorporated into a well-stirred, parallel-tube or other liver metabolism model [11]. Application of the IVIVE approach within the physiologically based pharmacokinetic (PBPK) models enables accounting for inter- and intra-individual variability in anatomy and physiology, such as changes in transporters and metabolizing enzyme abundance or function, in the estimation of human hepatic clearance and therefore in systemic and tissue concentrations of the drug. A range of scaling and correction factors, considering the differences in organ size, blood flow rates, or enzyme expression between the *in vitro* system and the human body, are used to bridge the gap between *in vitro* and *in vivo* drug behaviour. One of the critically important but often neglected [12] factor deciding on the accuracy and reliability of clearance IVIVE is drug binding occurring in the *in vitro* incubation [13–17].

The extent of binding is defined by the fraction of the unbound compound in the incubation (fu; 0%–100%). According to the Free Drug Theory, only an unbound fraction of the drug is available for tissue uptake, can cause the pharmacodynamic effect, and undergo metabolic changes [18, 19]. Going further, a good understanding of a specific and nonspecific binding to the proteins in the circulatory system or single cells is crucial for reliable prediction of the safety and effectiveness of the drug. Fu is used not only in the field of drug discovery and development but is also relevant in clinical practice, e.g. in safety assessment of the pharmacotherapy during breastfeeding [20] or risk of adverse reaction of the highly binding drugs in patients with hypoalbuminaemia [21]. Although the fu parameter is primarily perceived from the perspective of free fraction in plasma, fu in other body compartments or systems is no less important. For reliable pharmacokinetic–pharmacodynamic analysis and clinical dose assessment, knowledge of free drug concentration at the specific site of action is inevitable. For many compounds, especially those characterized by good permeability, in a steady state, free concentration in plasma is a good approximation of free concentration in tissues. However, in some cases, e.g. involvement of transporters or pH gradients, this assumption may not hold true [22, 23]. The discrepancy between fu *in vitro* incubations used to get data for human metabolism predictions and fu in plasma is even more pronounced and expected. Compound’s binding to proteins, labware, cell-attachment matrices, or partition into the lipid membrane [12, 24] can underlie the underprediction of the real clearance value, and it is widely accepted that accounting for incubational binding enhances the confidence in human clearance and PK *in vitro* prediction [25, 26]. Consequently, for the further use of the study results, it is beneficial to measure the fu. The main techniques that can be implemented to measure fu in various systems are equilibrium dialysis, ultrafiltration, and ultracentrifugation [27]. Although some publicly available databases of chemicals, such as PubChem or ChEMBL, provide some information about fu, these data are primarily focused on fu in plasma.

The aim of this work was to provide a detailed repository of the literature data on compound’s fu value in various *in vitro* systems used for drugs metabolism evaluation and corresponding human plasma binding level. The combination of data on fu measured in human and animal plasma, isolated hepatocytes, isolated microsomes, and recombinant Cytochromes P450 (CYPs) can be utilized for a detailed study on the impact of the methods used for experimental measurement on the accuracy of clearance prediction.

## Methods

To collect free drug concentration data, the Medline and ScienceDirect bibliographic databases were used together with the publicly available Google Scholar search engine for scientific publications. Databases were queried without a time limit. The following key phrases were used to build queries: ‘fraction unbound’, ‘plasma protein binding’, ‘free fraction’, ‘nonspecific binding’, ‘non-specific binding’, ‘microsomes’, ‘hepatocytes’, ‘incubation’, ‘metabolic clearance’, ‘recombinant enzymes’, ‘rhCYPs’, ‘metabolism’, ‘incubation’, and ‘in vitro system’. The mentioned keywords could appear in the title, in the content of the abstract, or main text of the publications. If the publication included supplementary data, these were also examined for the presence of the key terms. In the first step, fu values for three different *in vitro* incubations, i.e. hepatocytes, microsomes, or recombinant CYPs, were searched, and then for each drug having at least one value of free fraction measured in any *in vitro* system, the value of free fraction in human plasma was retrieved. If human plasma data were not found, other animal data were retrieved. The search was not limited to drug-like chemicals.

Every publication available in English has been carefully reviewed for eligibility. Only experimentally measured fu values were collected, and results obtained through calculations based on QSAR (Quantitative Structure-Activity Relationship) models using the physicochemical properties of a given drug were rejected. For all available publications that were not the original source of the value of a searched parameter, the primary source articles were found and cited.

The collected data were documented in a Microsoft Excel spreadsheet. The values of fraction unbound are presented as the mean and standard deviation (SD) of all retrieved experimental values for a given compound. For compounds for which the fu range was reported without mean value, it was placed in a separate column named ‘fu range’. If the experimental value of the free fraction was imprecisely defined (as ‘above’ or ‘below’ a given value), it was also noted in the ‘range’ column.

Data on the free fraction in plasma and different *in vitro* models were supplemented with the following information: the experimental method used for the assessment of the degree of drug binding, protein or cell concentration in the incubation, and other experimental conditions, if different from the standard ones, species, reference to the source publication, and the author’s name and date of publication.

## Results

In total, we collected 129 literature studies on 1425 different compounds Among these, 698 were defined by their chemical names. For these compounds, we added their simplified molecular-input line-entry system notation and information on the basic chemical properties: molecular weight, computationally estimated logarithm of the partition coefficient (XlogP), Topological Polar Surface Area, Hydrogen Bond Acceptor, and Hydrogen Bond Donor. Data were extracted from PubChem.

**Figure 2.**
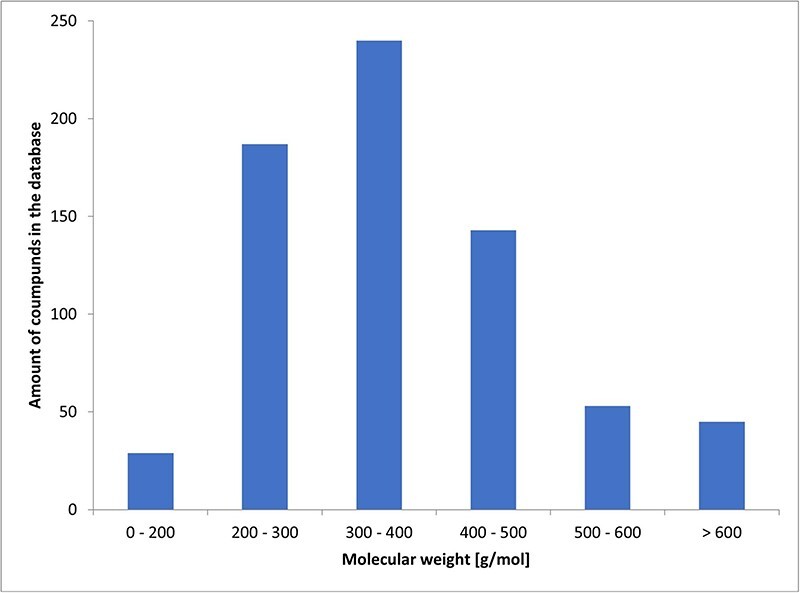
Histogram of molecular weights.

Among the known chemicals, molecular weight ranged from 129 to 1202 g/mol. The histogram illustrating the distribution of molecular weights is presented as Fig. 2.

**Figure 3.**
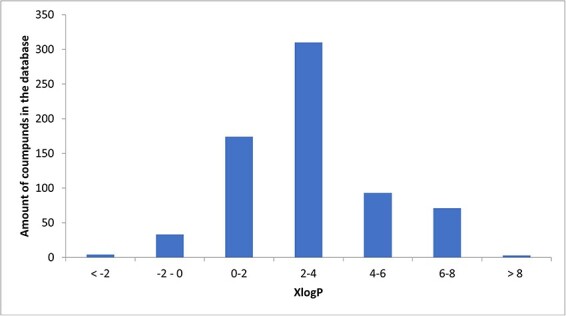
Histogram of XlogPs.

A total of 34 known compounds in the database were hydrophilic (XlogP < 0), 3 neutral (XlogP = 0), 651 lipophilic (XlogP > 0), and 81 highly lipophilic (XlogP ≥ 5). The histogram illustrating the distribution of XlogP is presented as Fig. 3.

For the fraction unbound in plasma, there are 2316 records available for 1349 unique compounds. Most of them (1327) were measured in human plasma, 622 in rats, 131 in dogs, 116 in nonhuman primates, 75 in mice, 3 in monkeys, and 2 in rabbits. For the remaining 40 records, species was not specified.

For the fraction unbound in hepatocytes, there are 863 records for 228 unique compounds. The number of records for human, rat, mouse, dog, and monkey was 394, 191, 94, 94, and 90, respectively.

For microsomes, we collected 2643 records for 1218 unique compounds. The number of records for human, rat, dog, nonhuman primate, monkey, mouse, Gottingen minipig, bama minipig, and rat was 1140, 670, 229, 115, 92, 60, 18, 11, and 2, respectively. For six records, the species was not specified in the source publication.

For the recombinant CYP enzymes, there are 61 records for 53 unique compounds, and there are 32, 13, 8, 3, 2, 2, 1 records for CYP 3A4, CYP 2D6, CYP 2C8, CYP 2A5, CYP 3A4, CYP 1A2, respectively.

The most commonly used method for measuring fu, regardless of the system, was equilibrium dialysis. Among the reported data for plasma, it was used in 87% of measurements, for hepatocytes in 52%, for microsomes in 83%, and for recombinant CYPs in 72%. However, for plasma and microsomal incubations, the experimental method for fu measurement was not specified in 55% (1292), and 2% (56) of records, respectively.

## Conclusions

In this work, we have collated a data set of experimentally measured values of fraction unbound for 1425 compounds, which includes data from three different *in vitro* systems and human plasma. The provided data set can be used as a reference for scientists involved in PK/PBPK modelling as well as researchers interested in QSAR models for the prediction of fraction unbound based on compound structure.

We observed that for the recombinant CYP *in vitro* systems, reporting of fraction unbound in the incubation is scarce as compared to other systems. This adds additional uncertainty to rCYP-based clearance predictions. Thus further research and efforts to provide more data on fu_rhCYP_ would be valuable, as accounting for binding is crucial for the reliability and appropriateness of *In Vitro*-*In Vivo* Correlation/*In Vitro*-*In Vivo* Extrapolation results for clearance [28].

## Supplementary Material

baae063_Supp

## Data Availability

Database is freely available under the open-access license at Mendeley, at https://tox-portal.com/#/, and as a Supplementary file.

## References

[1] Leelananda SP , Lindert S. Computational methods in drug discovery. *Beilstein J Org Chem* 2016;12:2694–718.doi: 10.3762/bjoc.12.267 28144341 PMC5238551

[2] Kim TH , Shin S, Shin BS. Model-based drug development: application of modeling and simulation in drug development. *J Pharm Investig* 2018;48:431–41.doi: 10.1007/s40005-017-0371-3

[3] Pantaleão SQ , Fernandes PO, Gonçalves JE et al. Recent advances in the prediction of pharmacokinetics properties in drug design studies: a review. *ChemMedChem* 2022;17:e202100542.doi: 10.1007/10.1002/cmdc.202100542 34655454

[4] Wu F , Zhou Y, Li L et al. Computational approaches in preclinical studies on drug discovery and development. *Front Chem* 2020;8:726.doi: 10.3389/fchem.2020.00726 PMC7517894 33062633

[5] Alqahtani S . In silico ADME-Tox modeling: progress and prospects. *Expert Opin Drug Metab Toxicol* 2017;13:1147–58.doi: 10.1080/17425255.2017.1389897 28988506

[6] Theil F-P , Guentert TW, Haddad S et al. Utility of physiologically based pharmacokinetic models to drug development and rational drug discovery candidate selection. *Toxicol Lett* 2003;138:29–49.doi: 10.1016/s0378-4274(02)00374-0 12559691

[7] Hallifax D , Foster JA, and Houston JB Prediction of human metabolic clearance from in vitro systems: retrospective analysis and prospective view. *Pharm Res* 2010;27:2150–61.doi: 10.1007/s11095-010-0218-3 20661765

[8] Lavé T , Coassolo P, and Reigner B. Prediction of hepatic metabolic clearance based on interspecies allometric scaling techniques and in vitro-in vivo correlations. *Clin Pharmacokinet* 1999;36:211–31.doi: 10.2165/00003088-199936030-00003 10223169

[9] Tess DA , Ryu S, and Di L. In vitro – in vivo extrapolation of hepatic clearance in preclinical species. *Pharm Res* 2022;39:1615–32.doi: 10.1007/s11095-022-03205-1 35257289

[10] Moreau M , Mallick P, Smeltz M et al. Considerations for improving metabolism predictions for in vitro to in vivo extrapolation. *Front Toxicol* 2022;4:894569.doi: 10.3389/ftox.2022.89456 PMC9099212 35573278

[11] Lerapetritou MG , Georgopoulos PG, Roth CM et al. Tissue-level modeling of xenobiotic metabolism in liver: an emerging tool for enabling clinical translational research. *Clin Transl Sci* 2009;2:228–37.doi: 10.1111/j.1752-8062.2009.00092.x 20443896 PMC3068531

[12] Gouliarmou V , Lostia AM, Coecke S et al. Establishing a systematic framework to characterise in vitro methods for human hepatic metabolic clearance. *Toxicol In Vitro* 2018;53:233–44.doi: 10.1016/j.tiv.2018.08.004 30099088 PMC10288526

[13] Gardner I , Xu M, Han C et al. Non-specific binding of compounds in in vitro metabolism assays: a comparison of microsomal and hepatocyte binding in different species and an assessment of the accuracy of prediction models. *Xenobiotica* 2022;52:943–56.doi: 10.1080/00498254.2022.2132426 36222269

[14] Deshmukh SV , and Harsch A. Direct determination of the ratio of unbound fraction in plasma to unbound fraction in microsomal system (fu p/fu mic) for refined prediction of phase I mediated metabolic hepatic clearance. *J Pharmacol Toxicol Methods* 2011;63: 35–39.doi: 10.1016/j.vascn.2010.04.003 20433934

[15] Obach RS . The importance of nonspecific binding in in vitro matrices, its impact on enzyme kinetic studies of drug metabolism reactions, and implications for in vitro-in vivo correlations. *Drug Metab Dispos* 1996;24:1047–49. 8894503

[16] Obach RS . Nonspecific binding to microsomes: impact on scale-up of in vitro intrinsic clearance to hepatic clearance as assessed through examination of warfarin, imipramine, and propranolol. *Drug Metab Dispos* 1997;25:1359–69. 9394025

[17] Obach RS . Prediction of human clearance of twenty-nine drugs from hepatic microsomal intrinsic clearance data: an examination of in vitro half-life approach and nonspecific binding to microsomes. *Drug Metab Dispos* 1999;27:1350–59. 10534321

[18] Summerfield SG , Yates JWT, Fairman DA. Free drug theory – no longer just a hypothesis? *Pharm Res* 2022;39:213–22.doi: 10.1007/s11095-022-03172-7 35112229

[19] Wanat K . Biological barriers, and the influence of protein binding on the passage of drugs across them. *Mol Biol Rep* 2020;47:3221–31.doi: 10.1007/s11033-020-05361-2 32140957

[20] Abduljalil K , Pansari A, Ning J et al. Prediction of drug concentrations in milk during breastfeeding, integrating predictive algorithms within a physiologically‐based pharmacokinetic model. *CPT Pharmacom & Syst Pharma* 2021;10:878–89.doi: 10.1002/psp4.12662 PMC8376129 34213088

[21] Roberts JA , Pea F, Lipman J. The clinical relevance of plasma protein binding changes. *Clin Pharmacokinet* 2013;52:1–8.doi: 10.1007/s40262-012-0018-5 23150213

[22] Ryu S , Tess D, Chang G et al. Evaluation of fraction unbound across 7 tissues of 5 species. *J Pharmaceut Sci* 2020;109:1178–90.doi: 10.1016/j.xphs.2019.10.060 31704191

[23] Zhang D , Hop CECA, Patilea-Vrana G et al. Drug concentration asymmetry in tissues and plasma for small molecule–related therapeutic modalities. *Drug Metab Dispos* 2019;47:1122–35.doi: 10.1124/dmd.119.086744 31266753 PMC6756291

[24] Nagar S , Korzekwa K. Drug distribution. Part 1. models to predict membrane partitioning. *Pharm Res* 2017;34:535–43.doi: 10.1007/s11095-016-2085-z 27981450 PMC5588161

[25] Chiba M , Ishii Y, Sugiyama Y. Prediction of hepatic clearance in human from in vitro data for successful drug development. *AAPS J* 2009;11:262–76.doi: 10.1208/s12248-009-9103-6 19408130 PMC2691463

[26] Jones RS , Leung C, Chang JH et al. Application of empirical scalars to enable early prediction of human hepatic clearance using IVIVE in drug discovery: an evaluation of 173 drugs. *Drug Metab Dispos* 2022;50:DMD-AR-2021-000784.doi: 10.1124/dmd.121.000784 35636770

[27] Dimitrijevic D , Fabian E, Funk-Weyer D et al. Rapid equilibrium dialysis, ultrafiltration or ultracentrifugation? Evaluation of methods to quantify the unbound fraction of substances in plasma. *Biochem Biophys Res Commun* 2023;651:114–20.doi: 10.1016/j.bbrc.2023.02.021 36812744

[28] Smith DA , Di L, and Kerns EH. The effect of plasma protein binding on in vivo efficacy: misconceptions in drug discovery. *Nat Rev Drug Discov* 2010;9:929–39.doi: 10.1038/nrd3287 21119731

